# Correction: Impact of gender on the formation and outcome of formal mentoring relationships in the life sciences

**DOI:** 10.1371/journal.pbio.3001859

**Published:** 2022-10-14

**Authors:** The PLOS Biology Staff

S3 Table was published with corrective annotations. Please view the correct version of S3 Table here. The publisher apologizes for the error.

**S3 Table:** Photo validation of automated gender estimates. For each human-labeled category (rows), right columns indicate classification provided by genderize.io. The data and code needed to generate this table are available on Zenodo (DOI: 10.5281/zenodo.4722020).

Classifier performance			
Category	N	# and % men	# and % women
Unknown	48 (2.7%)	40 (83.3%)	8 (16.7%)
Man	1542 (86.2%)	1539 (99.8%)	3 (0.2%)
Woman	198 (11.1%)	20 (10.1%)	178 (89.9%)

## References

[1] Schwartz LP, Liénard JF, David SV (2022) Impact of gender on the formation and outcome of formal mentoring relationships in the life sciences. PLoS Biol 20(9): e3001771. 10.1371/journal.pbio.3001771 36074782 PMC9455859

