# Supplementary figures and images for: Impact of gender on the formation and outcome of formal mentoring relationships in the life sciences
Source: PLoS Biol. 2022 Sep 8;20(9):e3001771. doi: 10.1371/journal.pbio.3001771 (PMC9455859; doi:10.1371/journal.pbio.3001771)

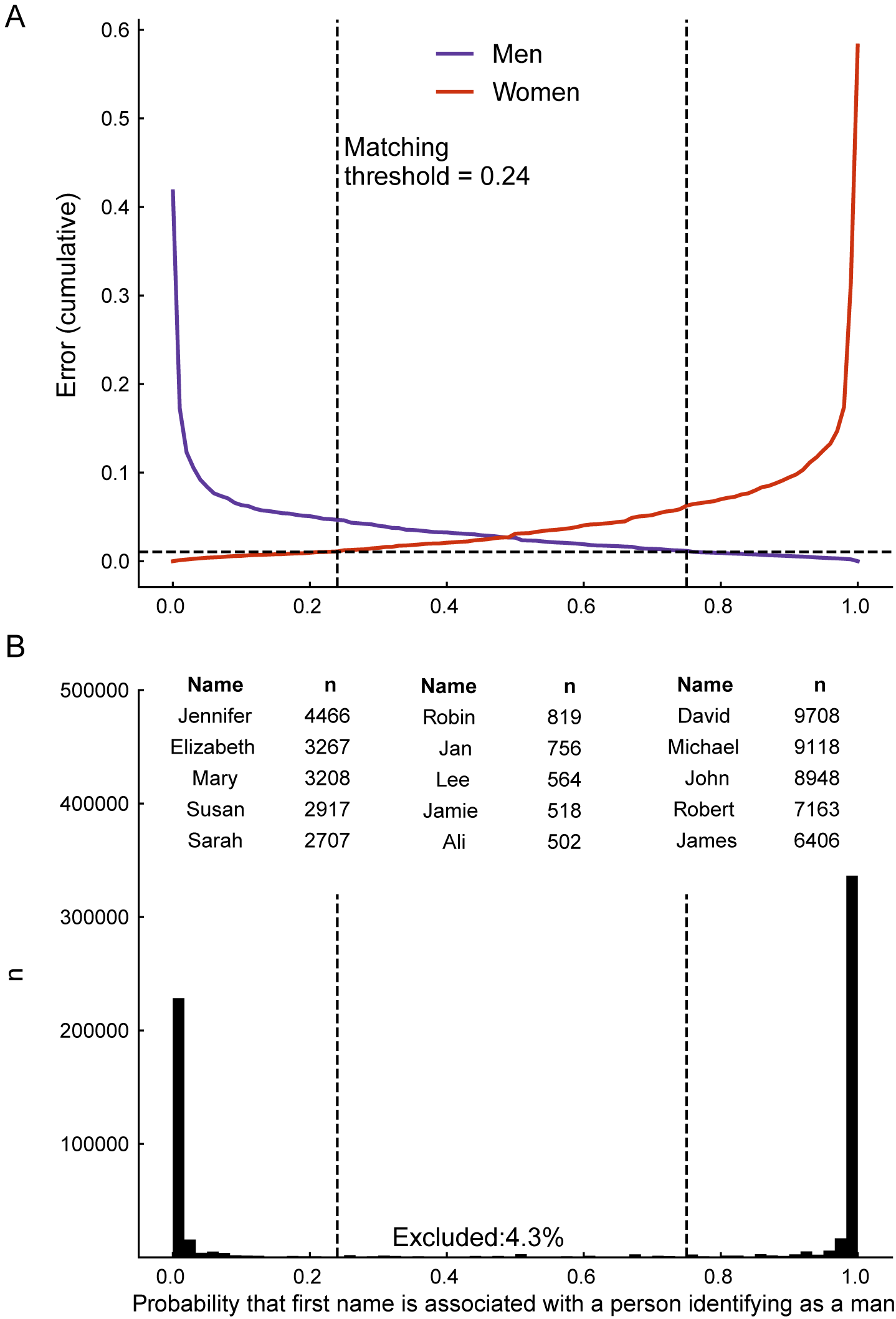

Supplement: S1 Fig — (A) Dashed lines indicate upper and lower thresholds for ambiguous first names. The threshold producing balanced error is slightly more conservative for women’s names (0.24) than for men’s (0.75). (B) Distribution of inferred genders. Lists at top indicate 5 most common names in the dataset in each category (probable man, ambiguous, and probable woman). The data and code needed to generate this figure are available on Zenodo (DOI: 10.5281/zenodo.4722020). (TIF) [file pbio.3001771.s001.tif]

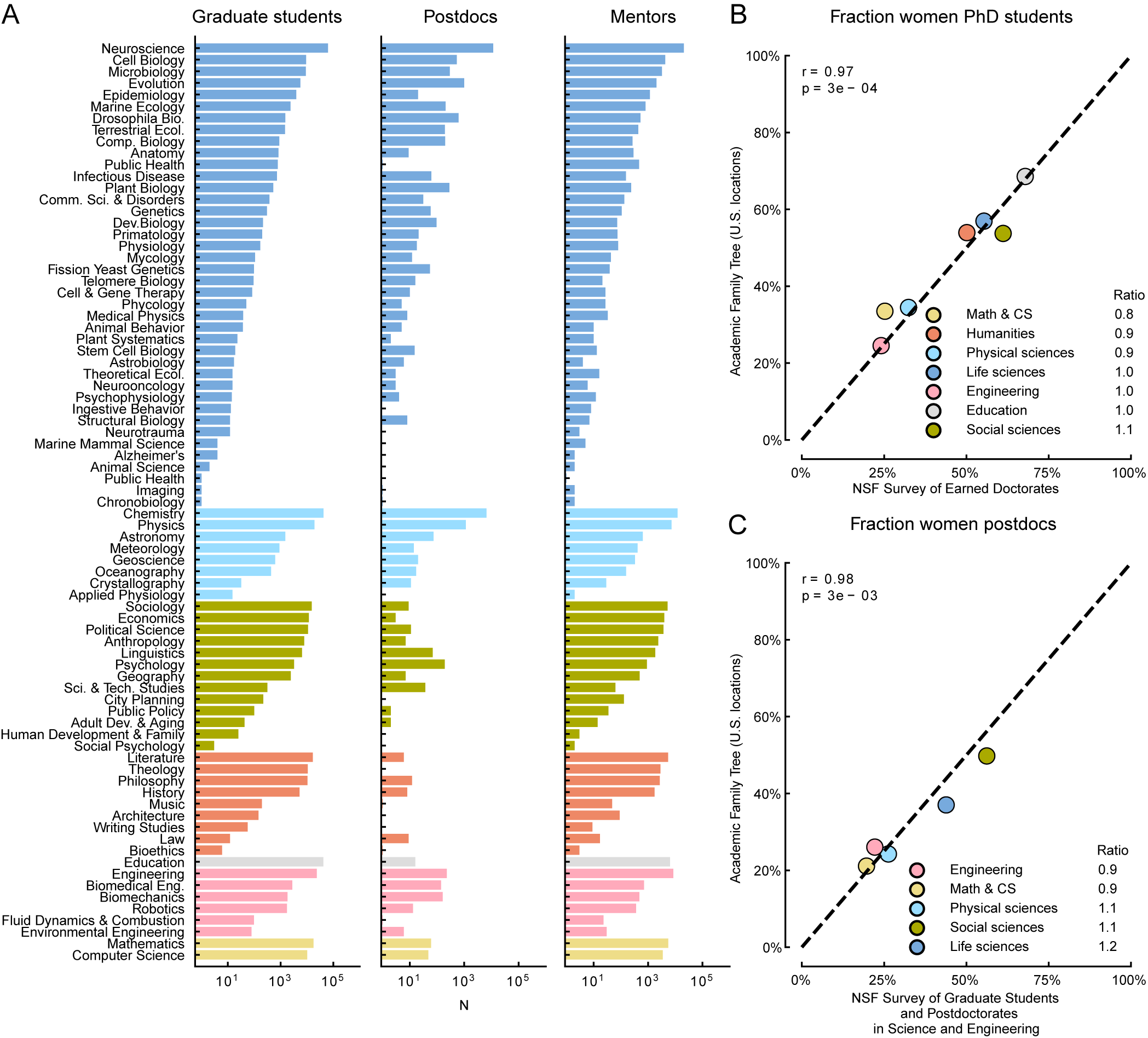

Supplement: S2 Fig — (A) Total graduate students, postdocs, and mentors in the AFT, during 2000–2020 and grouped by subfield. (B) Percentage of women graduate students in the AFT and the NSF’s Survey of Earned Doctorates. Each point represents data for 2000–2020 in one broad research field. Table shows ratio difference between the 2 datasets, (NSF/AFT, absolute difference across all research areas: +0.5%). (C) Percentage of women postdocs in the AFT and the NSF’s Statistical Survey of Graduate Students and Postdoctorates in Science and Engineering. Each point represents data for postdocs with training end dates between 2000–2020 in one broad research field (AFT data) or the 2015 cross-section of actively employed postdocs (NSF data). The data and code needed to generate this figure are available on Zenodo (DOI: 10.5281/zenodo.4722020). AFT, Academic Family Tree; NSF, National Science Foundation. (TIF) [file pbio.3001771.s002.tif]

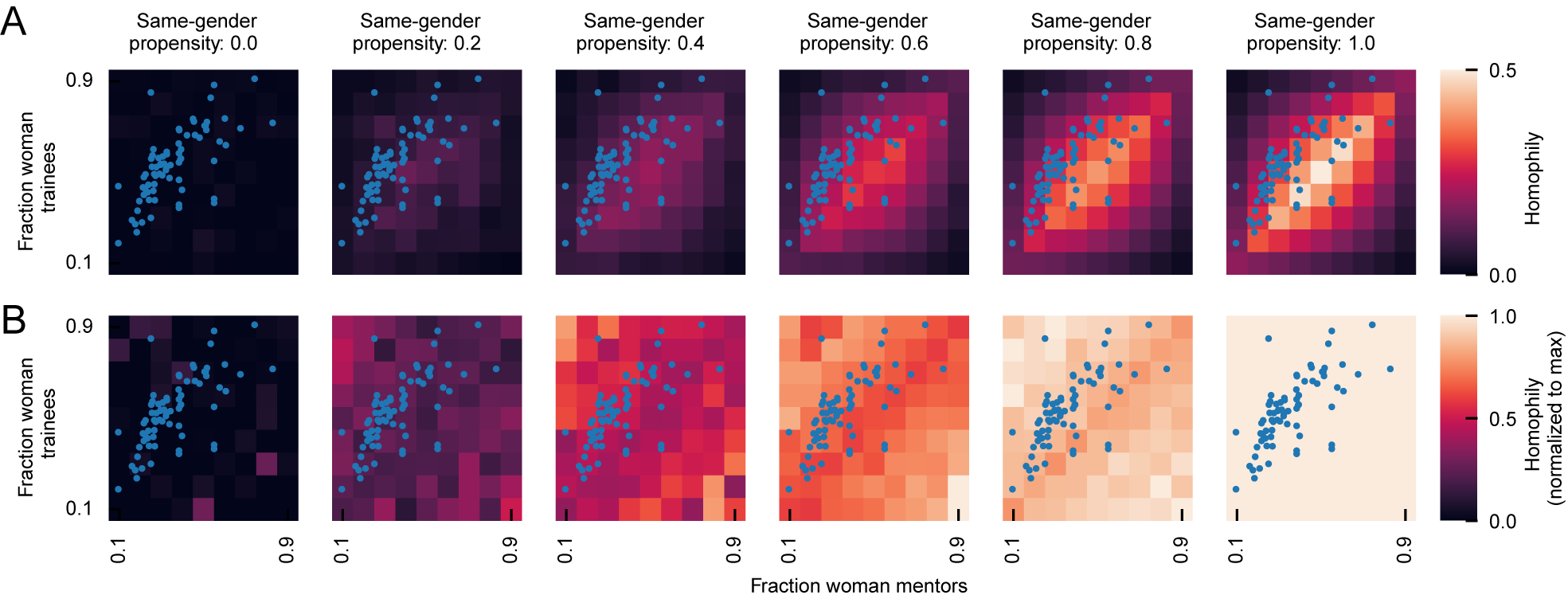

Supplement: S3 Fig — (A) Simulation of how measurements of gender homophily are affected by the gender composition of mentor and trainee pool within a field (see Methods). Each heatmap shows simulations for a different propensity for mentors and trainees to form same-gender pairs, ranging from 0 (none) to 1 (maximum). Scatter plot shows actual mentor and trainee gender composition for data used in the homophily analysis. Each point represents average composition for one narrow research field. When mentors or trainees of one gender are scarce, homophily does not reflect the underlying propensity to form same-gender pairs. (B) Simulation, after correcting for effects of gender composition of pool, with actual average compositions overlaid, as in (A). The data and code needed to generate this figure are available on Zenodo (DOI: 10.5281/zenodo.4722020). (TIF) [file pbio.3001771.s003.tif]

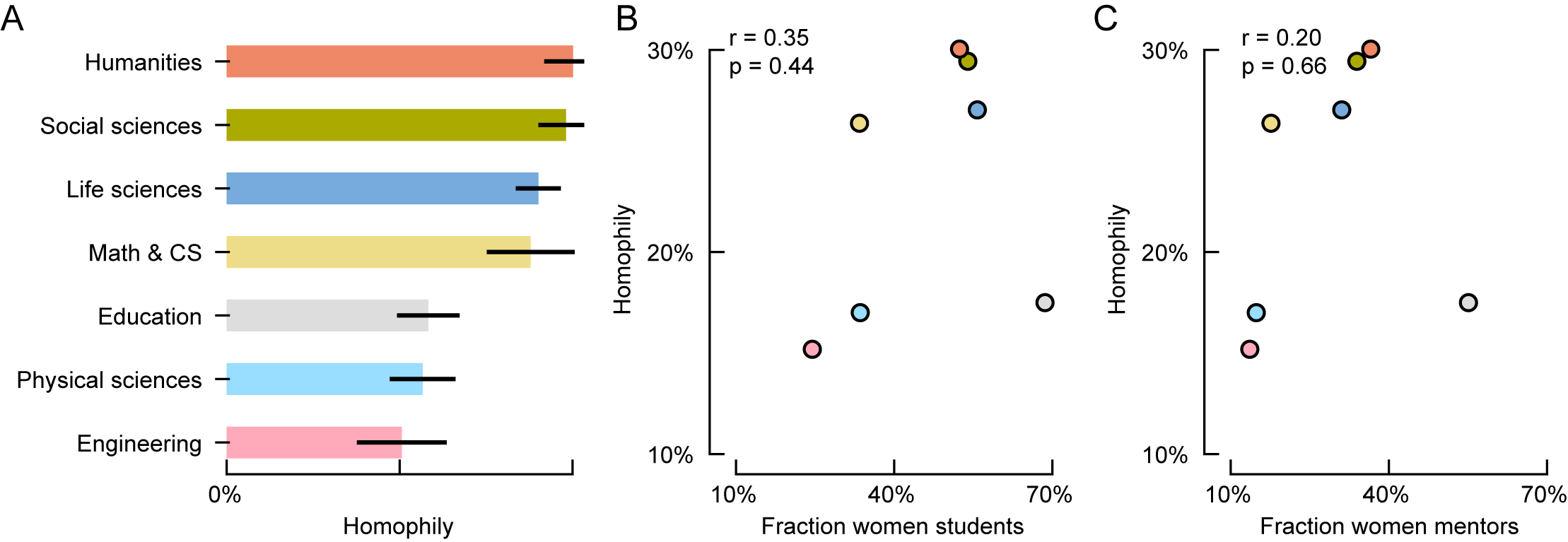

Supplement: S4 Fig — (A) Bars indicate homophily in each general research area. Error bars indicate bootstrapped 95% confidence intervals. (B) Scatter plot compares fraction women students versus homophily in each general research area. (C) Fraction women mentors versus homophily, as in (B). The data and code needed to generate this figure are available on Zenodo (DOI: 10.5281/zenodo.4722020). (TIF) [file pbio.3001771.s004.tif]

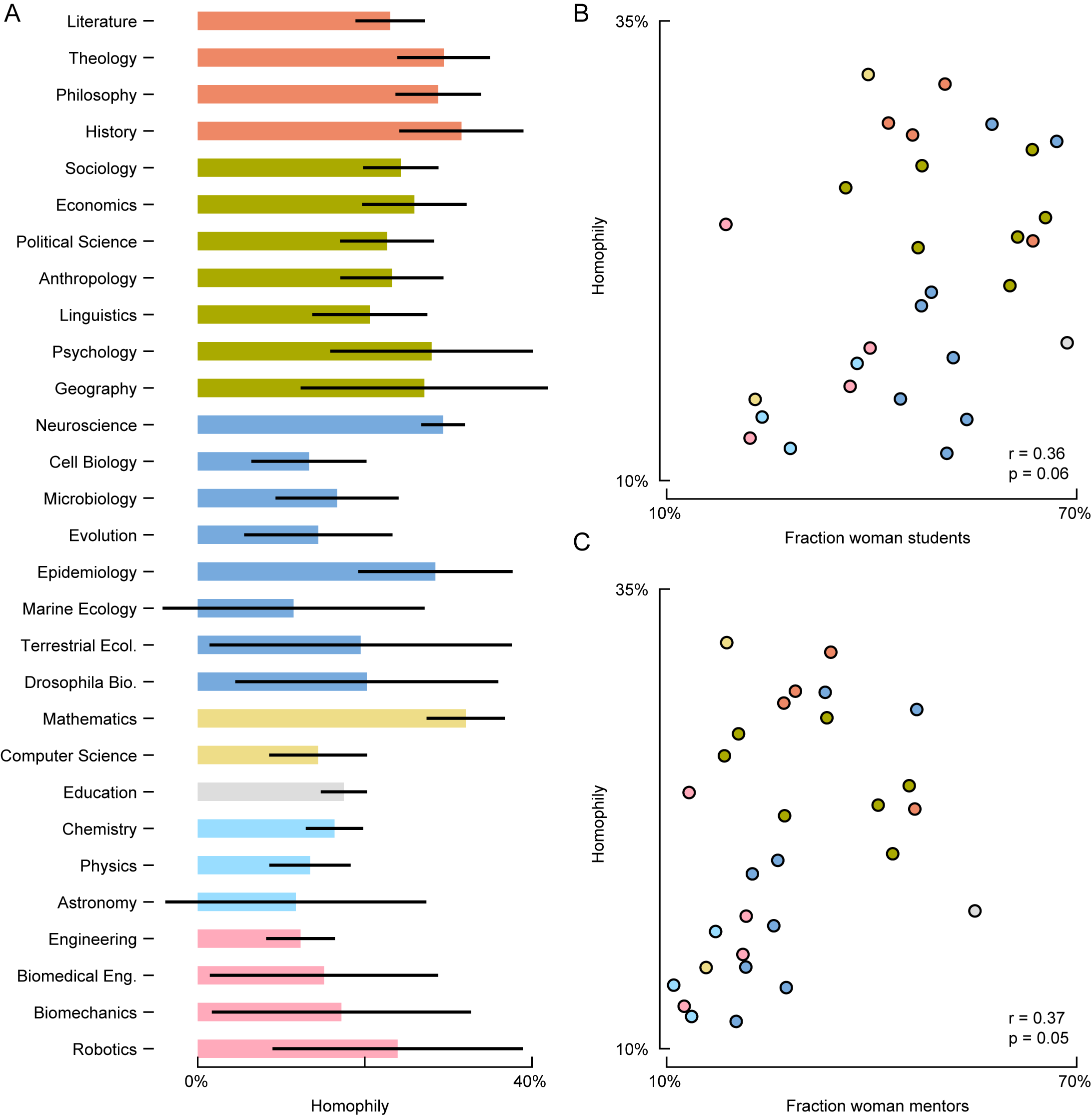

Supplement: S5 Fig — (A) Bars indicate homophily in narrow research areas with greater than 1,000 students. Error bars indicate bootstrapped 95% confidence intervals. (B) Scatter plot compares fraction women students versus homophily in each narrow research area. (C) Fraction women mentors versus homophily, as in (B). The data and code needed to generate this figure are available on Zenodo (DOI: 10.5281/zenodo.4722020). (TIF) [file pbio.3001771.s005.tif]

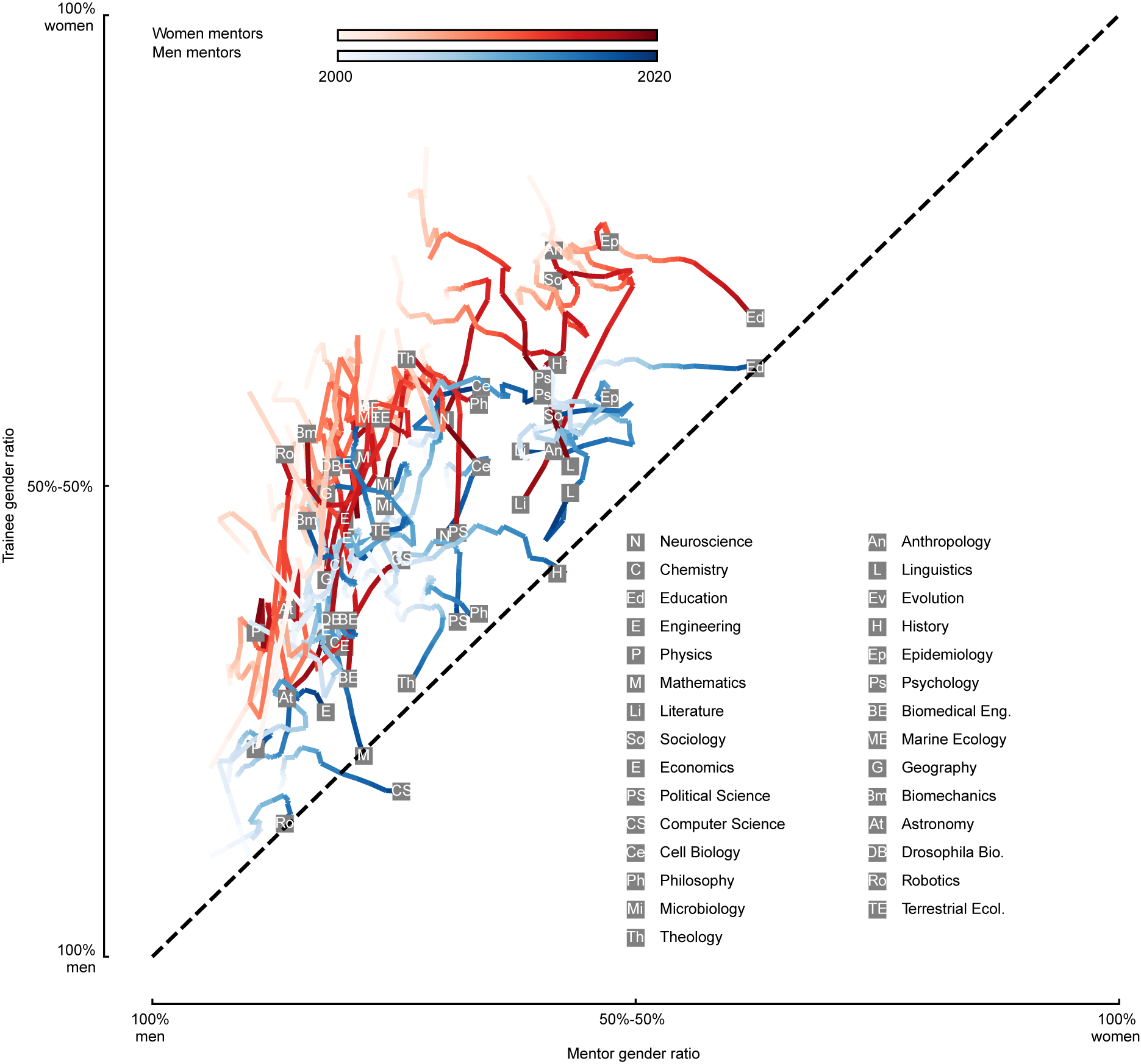

Supplement: S6 Fig — Colors indicate mentors’ gender and time of graduation. Field abbreviations are reported in the key at lower right. The consistent pattern of homophily is reflected in the greater fraction of women trainees of women mentors (red), shifted up relative to trainees of men mentors (blue). The data and code needed to generate this figure are available on Zenodo (DOI: 10.5281/zenodo.4722020). (TIF) [file pbio.3001771.s006.tif]

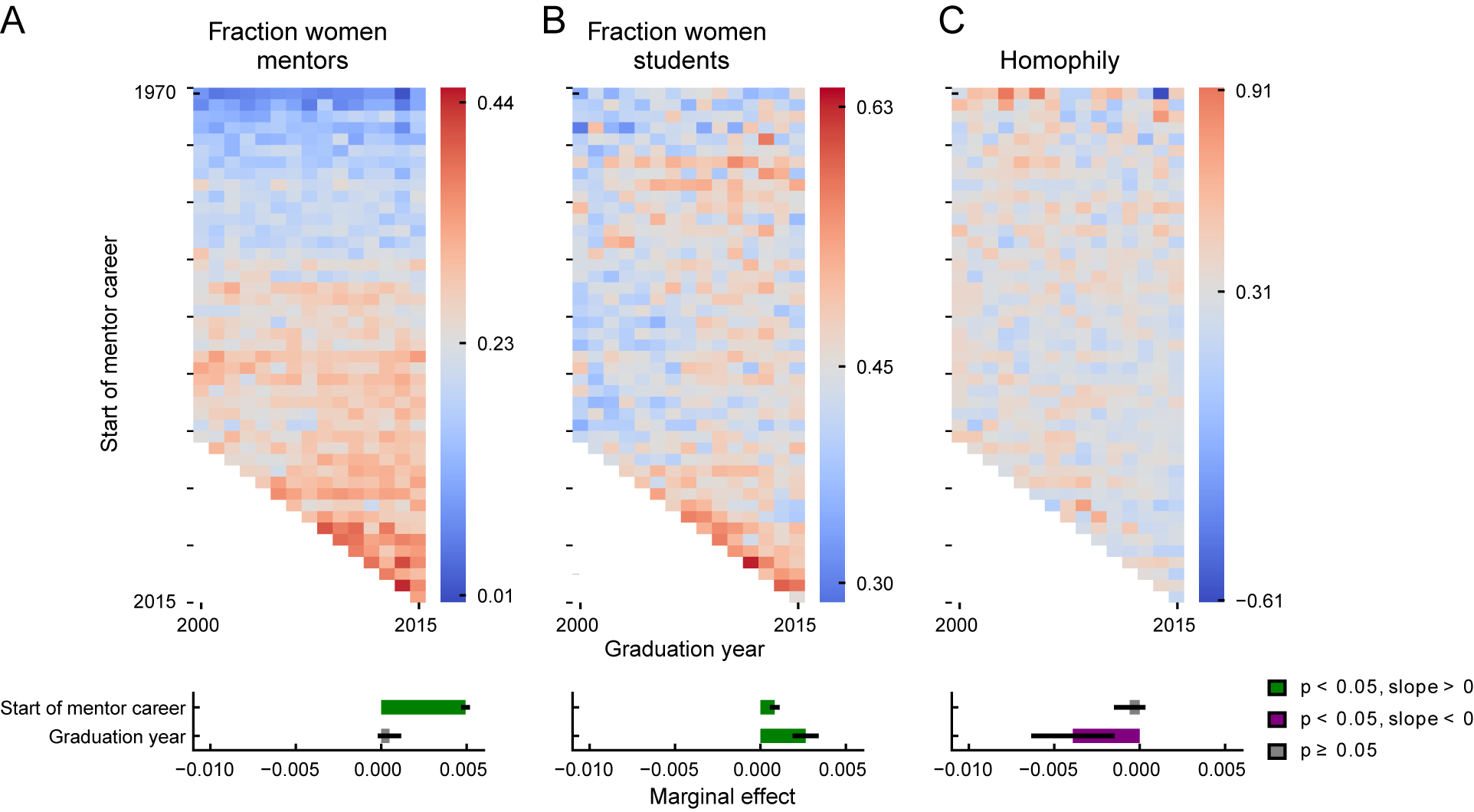

Supplement: S7 Fig — Heatmaps show (A) fraction women mentors, (B) fraction women students, and (C) homophily, grouped by year that mentor began independent career and year of student’s graduation (n = 37,692 mentors, 163,840 students). Bars at bottom indicate results of multivariate linear regression predicting the gender-related variable from both temporal variables. Error bars indicate 95% confidence intervals. The data and code needed to generate this figure are available on Zenodo (DOI: 10.5281/zenodo.4722020). (TIF) [file pbio.3001771.s007.tif]

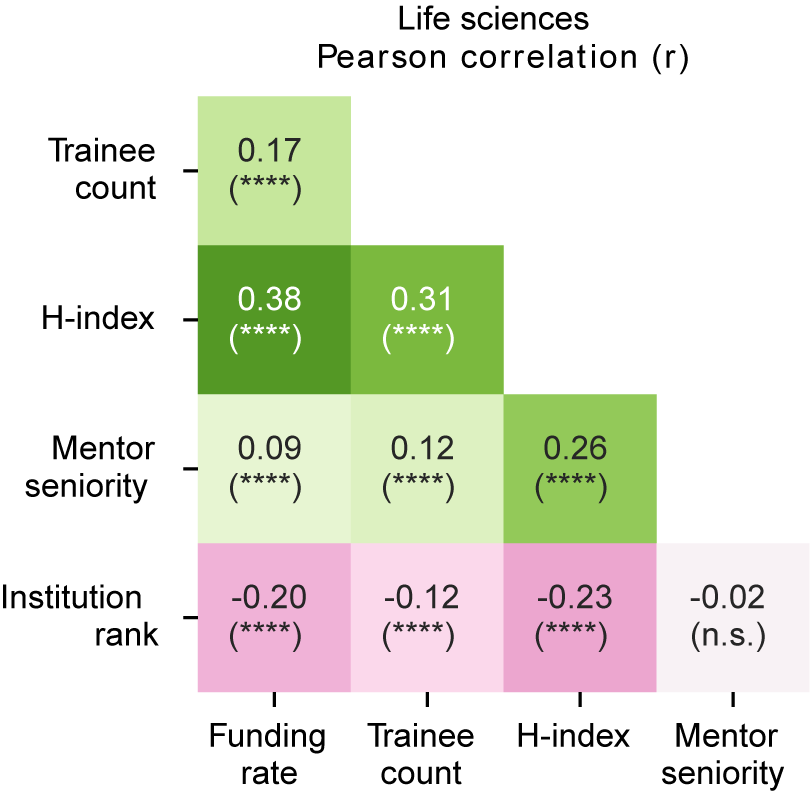

Supplement: S8 Fig — ****: p<0.0001, n.s.: not significant, p≥0.05. The data and code needed to generate this figure are available on Zenodo (DOI: 10.5281/zenodo.4722020). (TIF) [file pbio.3001771.s008.tif]

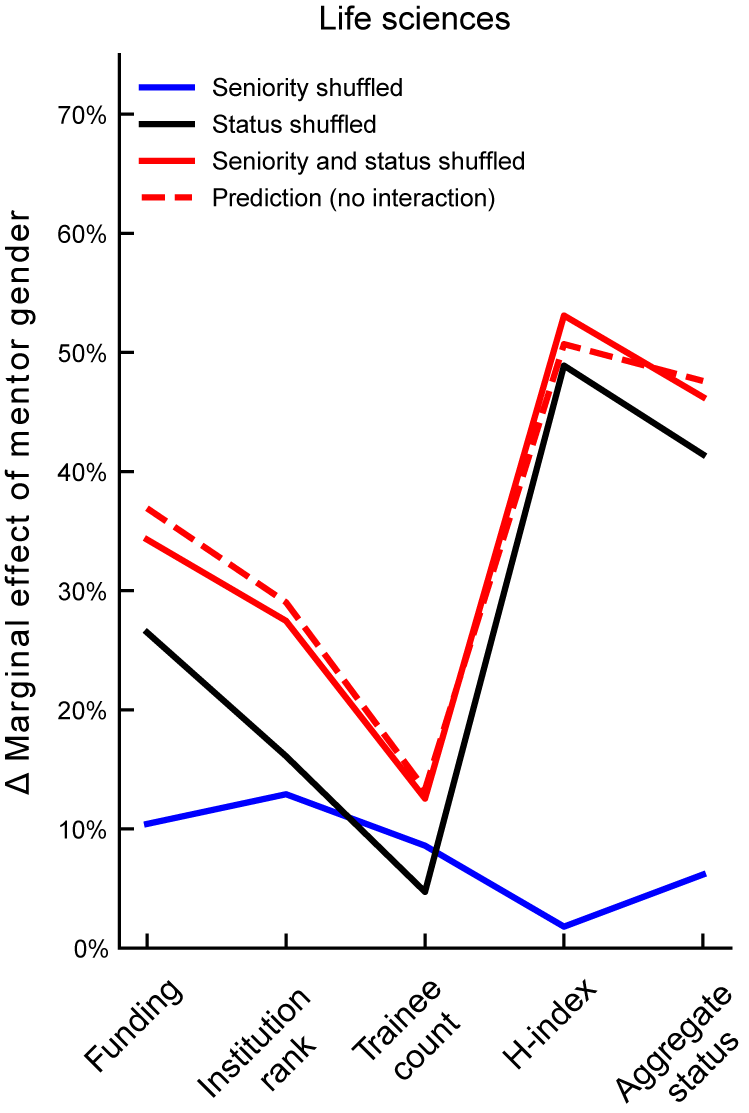

Supplement: S9 Fig — Dashed line indicates prediction of reduction for no interaction between status and seniority (i.e., the sum of the reduction from randomizing seniority and randomizing status). The data and code needed to generate this figure are available on Zenodo (DOI: 10.5281/zenodo.4722020). (TIF) [file pbio.3001771.s009.tif]

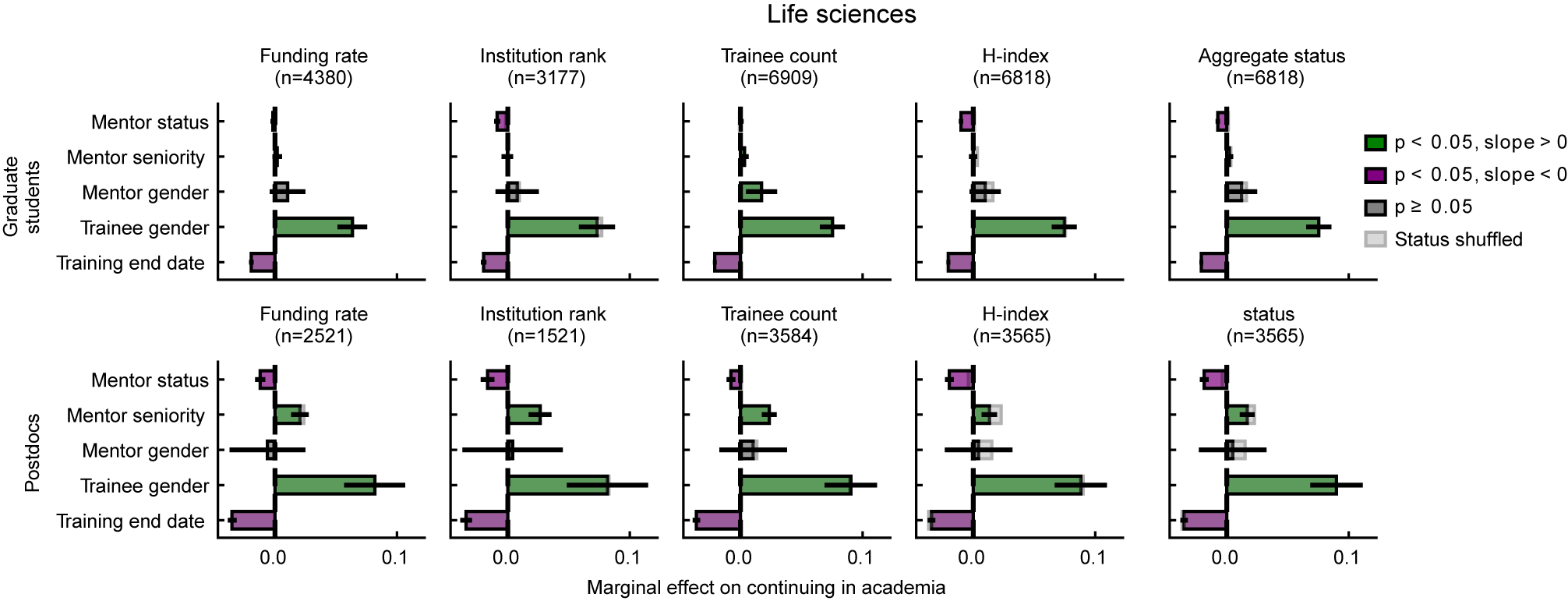

Supplement: S10 Fig — Marginal effects predicted by logistic regression model on individual trainee’s continuation to mentorship based on mentor status, mentor and trainee gender, and training end date, fit separately to data for postdocs and graduate students. The data and code needed to generate this figure are available on Zenodo (DOI: 10.5281/zenodo.4722020). (TIF) [file pbio.3001771.s010.tif]

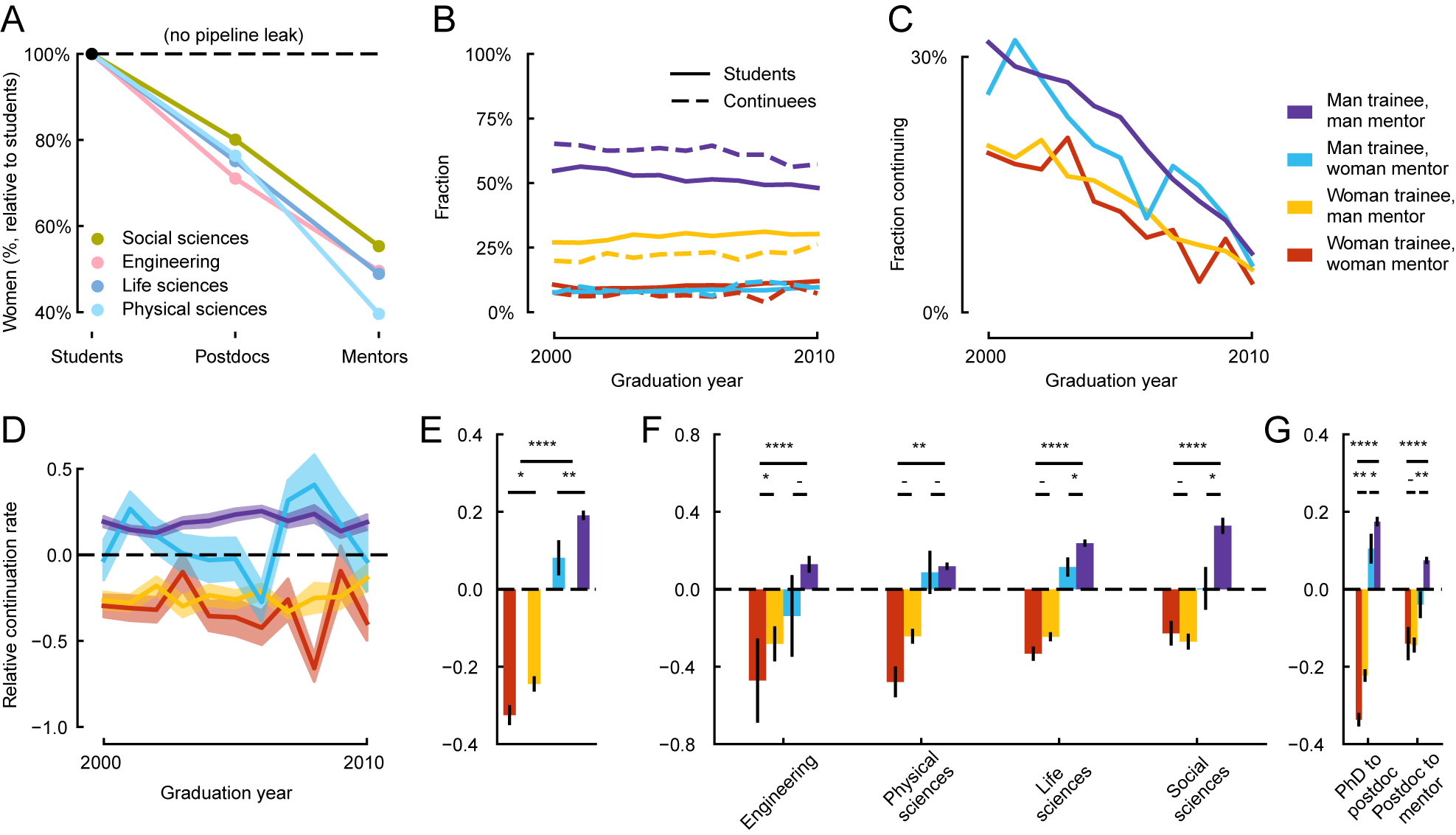

Supplement: S11 Fig — (A) Fraction of women postdocs and mentors within fields, relative to fraction of women graduate students in the field. (B) Proportion of graduate students in each trainee-gender/mentor-gender group across all fields, and proportion that continue to academic mentorship roles. (C) Fraction of graduate students within each group that continued to mentorship roles. (D-G) Mean difference between continuation rates for each gender group and the overall continuation rate in a given year. Error bars show jackknifed standard error. Stars indicate significance of trainee gender (top row) or mentor gender (lower row) weights in logistic regression predicting continuation based on stop year, trainee gender, and mentor gender (****: p<0.0001, ***: p<0.001, **: p<0.01, *: p<0.05, n.s.: p≥0.05). The data and code needed to generate this figure are available on Zenodo (DOI: 10.5281/zenodo.4722020). (TIF) [file pbio.3001771.s011.tif]
