# Supplementary material for: Impact of gender on the formation and outcome of formal mentoring relationships in the life sciences
Source: PLoS Biol. 2022 Sep 8;20(9):e3001771. doi: 10.1371/journal.pbio.3001771 (PMC9455859; doi:10.1371/journal.pbio.3001771)
Supplement: S1 Table — Slope indicates annual change in homophily from 2000–2020, based on linear regression predicting homophily by year. Asterisks indicate significance of temporal effect *:p<0.05, **: p<0.01, ***: p<0.001. The data and code needed to generate this table are available on Zenodo (DOI: 10.5281/zenodo.4722020). (PDF) [file pbio.3001771.s012.pdf]

Field	Students		Mentors		Homophily	
	n	% women	n	% women	%	Slope
Anthropology	7959	61	2354	41	23	0.010
Astronomy	1569	28	622	14	12	-0.010
Biomechanics	1858	40	462	22	17	-0.005
Biomedical Eng.	2768	37	685	21	15	0.000
Cell Biology	9248	54	4184	28	13	-0.004
Chemistry	43027	38	11198	17	16	-0.007 (***)
Computer Science	10245	23	3447	16	14	-0.013 (**)
Drosophila Bio.	1543	49	453	26	20	0.001
Economics	11818	36	3949	18	26	-0.020 (****)
Education	41748	69	6384	55	17	-0.017 (****)
Engineering	23692	22	8445	13	12	-0.004
Epidemiology	3948	67	1161	47	28	0.010
Evolution	5978	44	1848	22	14	0.000
Geography	2432	47	492	21	27	-0.003
History	5360	51	1731	34	32	-0.021 (***)
Linguistics	6733	60	1806	43	21	-0.012 (*)
Literature	16660	64	5478	46	23	0.004
Marine Ecology	2557	51	763	20	11	-0.024
Mathematics	17582	40	5521	19	32	0.001
Microbiology	9117	52	3185	26	17	-0.025 (*)
Neuroscience	66153	58	19218	33	29	-0.008 (***)
Philosophy	10511	46	2681	27	29	0.010
Physics	19542	24	7008	11	13	0.003
Political Science	11067	47	3682	27	23	-0.028 (*)
Psychology	3384	64	855	33	28	-0.022 (***)
Robotics	1740	19	361	13	24	-0.006
Sociology	15287	65	5183	45	24	0.012
Terrestrial Ecol.	1546	47	408	23	20	-0.009
Theology	10665	42	2843	29	29	-0.006

Table S1. Homophily across narrow research areas with at least 1000 students. Slope indicates annual change in homophily from 2000-2020, based on linear regression predicting homophily by year. Asterisks indicate significance of temporal effect *:$p < 0.05$, **:$p < 0.01$, ***:$p < 0.001$.
