# Supplementary material for: Impact of gender on the formation and outcome of formal mentoring relationships in the life sciences
Source: PLoS Biol. 2022 Sep 8;20(9):e3001771. doi: 10.1371/journal.pbio.3001771 (PMC9455859; doi:10.1371/journal.pbio.3001771)
Supplement: S2 Table — Each row shows median gender homophily across all fields and years, marginal effect of trainee and mentor gender on trainee continuation to mentorship (see Fig 3), and change in the effect of mentor gender after controlling for mentor aggregate status for a single threshold. Threshold refers to the minimum/maximum probability of a first name identifying a man for classification as a man/woman. Excluded indicates the percent of individual excluded for having ambiguous names according to that threshold. For all thresholds, effects of mentor gender were not significant in models that accounted for status (see Fig 4). **: p<0.0001, *: p<0.05. Data using alternative thresholds are available from the authors on request. The code needed to generate this table is available on Zenodo (DOI: 10.5281/zenodo.4722020). (PDF) [file pbio.3001771.s013.pdf]

| Threshold | | | | Status shuffled | | With status | | |
|---|---|---|---|---|---|---|---|---|
| Men | Women | Excluded | Median homophily | Trainee gender | Mentor gender | Trainee gender | Mentor gender | Mentor status correction |
| 0.60 | 0.36 | 1.8% | 19.4% | .102 (**) | .028 (**) | .100 (**) | 0.017 (*) | -41% |
| 0.75 | 0.24 | 4.3% | 20.5% | .103 (**) | .027 (**) | .100 (**) | 0.016 (*) | -41% |
| 0.9 | 0.09 | 7.6% | 21.0% | .105 (**) | .025 (**) | .102 (**) | 0.015 (*) | -42% |

**Table S2. Alternative thresholds for gender classification.** Each row shows median gender homophily across all fields and years, marginal effect of trainee and mentor gender on trainee continuation to mentorship (see Fig. 3), and change in the effect of mentor gender after controlling for mentor aggregate status for a single threshold. Threshold refers to the minimum/maximum probability of a first name identifying a man for classification as a man/woman. Excluded indicates the percent of individual excluded for having ambiguous names according to that threshold. For all thresholds, effects of mentor gender were not significant in models that accounted for status (see Fig. 4). **:$p < 0.0001$, *: $p < 0.05$.
