# Supplementary material for: Impact of gender on the formation and outcome of formal mentoring relationships in the life sciences
Source: PLoS Biol. 2022 Sep 8;20(9):e3001771. doi: 10.1371/journal.pbio.3001771 (PMC9455859; doi:10.1371/journal.pbio.3001771)
Supplement: S3 Table — For each human-labeled category (rows), right columns indicate classification provided by genderize.io. The data and code needed to generate this table are available on Zenodo (DOI: 10.5281/zenodo.4722020). (PDF) [file pbio.3001771.s014.pdf]

		Classifier performance	
Category	N	# and % men	# and % women
Unknown	~~46 (2.5~~48 (2.7%)	~~39 (85~~40 (83.3%)	~~7 (15~~8 (16.7%)
Man	~~1544 (86.4~~1542 (86.2%)	~~1540 (99.7~~1539 (99.8%)	~~4 (0.3~~3 (0.2%)
Woman	198 (~~11~~11.1%)	20 (10.1%)	178 (89.9%)

Table S3. Photo validation of automated gender estimates. For each human-labeled category (rows), right columns indicate classification provided by `genderize.io`.
