# Supplementary material for: Impact of gender on the formation and outcome of formal mentoring relationships in the life sciences
Source: PLoS Biol. 2022 Sep 8;20(9):e3001771. doi: 10.1371/journal.pbio.3001771 (PMC9455859; doi:10.1371/journal.pbio.3001771)
Supplement: S1 Appendix — (PDF) [file pbio.3001771.s015.pdf]

Impact of gender on the formation and outcome of formal mentoring relationships in the life sciences

Leah P. Schwartz[1], Jean F. Liénard[1], Stephen V. David[1*]

1 Oregon Hearing Research Center, Oregon Health and Science University, Portland, Oregon, United States of America

* davids@ohsu.edu

Appendix: Analysis of relative continuation rate between gender groups

The proportion of women in social science and STEM fields is lower at progressively later stages of the academic career track, from graduate student to postdoc to mentor (S11A Fig). Between 2000 and 2010, there was a small increase in the proportion of women graduate students (S11B Fig, 38% to 42%, $p = 0.006$) and women graduate mentors (18% to 22%, $p = 0.005$, linear regression predicting gender composition from year). However, the percentage of women students within each graduate cohort that went on to become academic mentors was consistently less than their proportion in the population of graduate students (S11B Fig). Despite a decreasing temporal trend in continuation rates for both men and women, the percentage of students that continued on to mentorship roles was consistently greater for men Ph.D. students, regardless of the gender of their mentor (S11C Fig).

To quantify effects of gender on continuation, we used the relative continuation rate, defined as the difference in continuation rate between members of one group (e.g., women students with women mentors) and the base rate of continuation across all groups. Continuation rate is defined as the ratio of continuees, c, to total trainees, n, in a group:

$$\Delta\text{group} = \frac{c_{\text{group}}/n_{\text{group}} - c_{\text{all}}/n_{\text{all}}}{c_{\text{all}}/n_{\text{all}}} \tag{1}$$

To control for decreases in the overall continuation rate over time, we calculated the relative continuation rate for each year, then took the mean (S11D-G Figs).

To assess the significance of mentor and trainee gender effects on continuation (S11E-G Figs), we fit logistic regression models predicting the probability that the trainee continued to an academic mentorship role (p) based on mentor and trainee gender and trainee's training end date ($year$):

$$log(\frac{p}{1-p}) = \beta_0 + \beta_1\text{year} + \beta_2\text{gender}_{\text{mentor}} + \beta_3\text{gender}_{\text{trainee}} + \epsilon \tag{2}$$

To assess the overall significance of trainee gender, we fit the following model:

$$log(\frac{p}{1-p}) = \beta_0 + \beta_1\text{year} + \beta_2\text{gender}_{\text{trainee}} + \epsilon \tag{3}$$

For analysis that separated out career stages (S11G Fig), Ph.D. students were considered to have continued to a postdoc if they had a post-doctoral training relationship in AFT. Postdocs were considered to have continued to mentorship if they had trainees listed in AFT.

Both trainee and mentor gender were associated with differences in continuation rate: across research areas and career stages, men students and students of men mentors continued to mentorship positions at rates greater than the base rate of continuation (S11D-G Figs). Differences in academic continuation related to trainee gender were greater in magnitude than those related to mentor gender, and reached statistical significance more consistently among subsets of the data. Associations between student gender and academic continuation were statistically significant in all research areas examined (engineering, physical, life, and social sciences, see S11F Fig). Associations between mentor gender and academic continuation were statistically significant only among men students in life sciences, the most densely sampled field of study in the dataset (S11F Fig). While not significant, other fields did all show a trend in the same direction.

The above data focused on the outcome of Ph.D. training only. However, associations between mentor and trainee gender and trainee continuation were present at both the transition from Ph.D. to postdoc and postdoc to mentor (S11G Fig). To test the joint influence of multiple mentors on trainee continuation, we identified the group of trainees with records of both graduate and postdoctoral training in Academic Family Tree, and fit logistic regression models that included temporal controls (training date and mentor seniority), trainee gender, and the gender of the graduate mentor, postdoctoral mentor, or both. Interestingly, in this case only the gender of the postdoctoral mentor was associated with training outcomes (n = 16907, beta = 0.261, p = 0.024 [postdoctoral mentor], beta = 0.121, p = 0.295 [graduate mentor]). We observed the same pattern if we focused only on life sciences data (n = 10526, beta = 0.374, p = 0.007 [postdoctoral mentor], beta = 0.159, p = 0.256 [graduate mentor]). This difference suggests that among trainees that continue to a postdoc, the gender of the postdoctoral mentor exerts a stronger influence on continuation than the gender of the graduate mentor.

While the gender composition of trainees and mentors changed during the 2000-2010 period (S11B Fig), the relative continuation rates of different trainee gender-mentor gender dyads did not change significantly (S11D Fig, $p > 0.05$ for all groups, linear regression predicting relative continuation rate from year). Thus, while the fraction of women graduate students and mentors increased during the decade studied, the data indicated no change in relative continuation rates in either group.
