# Supplementary material for: Impact of gender on the formation and outcome of formal mentoring relationships in the life sciences
Source: PLoS Biol. 2022 Sep 8;20(9):e3001771. doi: 10.1371/journal.pbio.3001771 (PMC9455859; doi:10.1371/journal.pbio.3001771)
Supplement: Attachment — Submitted filename: r2r_plos_biol.docx [file pbio.3001771.s016.docx]

This is a revised version of the manuscript PBIOLOGY-D-21-01377. We thank the reviewers for their thoughtful input. We appreciate in particular the concern that data around gender and scientific careers are complex and prone to misrepresentation if not analyzed and explained carefully. We considered these concerns carefully, and we hope the revised text adequately addresses them. Because these issues are of broad interest to the research community, we believe that our analysis of these issues will be of broad interest to the *PLoS Biology* readership.

Summary of major changes:

- We have substantially changed the language and structure of the manuscript to emphasize the hypothesis that structural factors drive differences in training outcomes correlated with mentor gender. Thanks to reviewer input we now use consistent language and avoid making simplistic statements that can be interpreted out of context as judging the quality of mentorship by men and women as different.
- The analysis characterizing the correlation in outcome between mentor gender and training outcome (original Fig. 2) has been moved to the supplemental material, so that readers will not accidentally interpret this finding as conclusive, rather than the more nuanced series of results that now appear in Figs. 2-4.
- We have focused out analysis of mentor status versus training outcomes on the biosciences, which represents the bulk of valid data in the AFT dataset. This removes concerns about bias for physical science and humanities fields, where less status data is available.

A point-by-point reply to specific reviewer concerns follows, with the original comments quoted in *italics*. Changes from the original submission are highlighted in a marked-up version of the manuscript.

**Reviewer #1:**

*This is a very interesting article discussing the role of gender homophily in mentor-mentee relationship. The findings suggested that (a) there is significant gender homophily in academic mentor-mentee relationship; (2) the gender homophily has impact on scientists' outcome, such as scientific impact, funding rate and etc. And this also has effect on the trainee's continuation in academia.*

Thank you. We agree that the interaction between homophily and gender differences in mentor status is an important one to consider when trying to understand the leaky pipeline for gender.

*I would recommend acceptance if authors can address the following points:*

***R1.1.*** *It's surprising that 3% trainer-trainee relationships are excluded when business and clinical medicine were removed (page 5). Therefore, I was wondering whether there are field differences when reporting trainer-trainee relationships.*

The Academic Family Tree (AFT) relies on two main sources for information on training relationships: crowdsourced data and ProQuest’s collection of dissertations and theses. We report field differences in the number of training relationships in the dataset in Fig. S2. We have added the following text to the Materials and Methods section to clarify possible field differences in reporting (lines 432-439):

There is a correlation between the fraction of Ph.D.-level training relationships in each broad field in the AFT dataset and concurrent data from the National Science Foundation’s census of Ph.D.-level training relationships (r=0.84, p=0.009, AFT data limited to training relationships at U.S. institutions). Thus, the sampling in the overall AFT dataset matches that reported by the NSF. Examining crowd-sourced data alone shows an overrepresentation of the life sciences and physical sciences relative to their proportion in NSF data. This trend may be due to the history of AFT, which began as an effort to track the academic genealogy of neuroscience through crowd-sourcing [21].

The low fraction of training relationships from business and clinical medicine that you have noted may also be due to the fact that a Ph.D. is not required for a career in these fields.

***R1.2.*** *IMHO, Figure 1A was not very straightforward. Presenting the relationship between mentor's gender ratio and mentee's gender ratio with trend is a bit confusing. I would suggest moving the trend information to the SI.*

Thank you for your input. We included the trend information because it demonstrates that gender homophily is consistently positive over time. Given that this point is also conveyed by Figure 1B, we have removed the trend lines and replaced Figure 1A with a scatterplot of the mentor vs. mentee gender ratios across all years. We have retained the original format for presenting trend information in the supplementary higher-resolution breakdown of fields (Fig. S6) because it offers a compact means of demonstrating the persistence of homophily over time for a large number of fields.

***R1.3.*** *In figure 3, given you present all fields together, you may need to normalize those measures within field, such as funding rate, trainee count, h-index and etc.*

We agree, and appreciate that we may not have been clear in the initial manuscript. We did in fact normalize status metrics within fields, though, this normalization had surprisingly little effect. Now that the current manuscript focuses on biosciences, which represents a large portion of the AFT dataset, the question of normalization is no longer relevant here.

***R1.4.*** *Beside analysis in figure 6, I would suggest a formal fixed-effect OLS where several confounding [variables] are included, for example scientists' topics.*

We agree that a more extensive model would help control for potential confounds. We have integrated a fixed-effects OLS including scientists’ award receipt into the referenced figure (Figure 5 in the revised manuscript). Because this analysis is now focused on life sciences, controlling for different fields is not necessary. The result is described on lines 237-259:

Higher status - as measured by funding rate, h-index, trainee count, institution rank, or the aggregate status metric - did not show a consistent relationship with the percentage of men students trained by men or women mentors (Fig. 5A). The degree of homophily, which collapsed across both men and women mentors, did not show a relationship with mentor status for any individual status metric or the aggregate success metric (Fig. 5B, p ≥ 0.05, linear regression predicting homophily from mentor status bin).

...

To more rigorously test the interaction between award receipt and the gender composition of student trainees, we turned to multivariate regression. A regression predicting fraction of men students for each life science mentor based on award receipt, mentor gender, and success rank showed an effect for award (β=0.077, p=2.1e-7) and mentor gender (β=0.16, p < 10−30, n=17205 mentors) but only a trend for status (β=-0.0017, p=0.057).

***R1.5.*** *In page 24, I don't think the null model take some important confounding variables into consideration, such as ethnicity, research interest, the trend of women increased participation in each field.*

We agree that women’s increased participation in science may affect measurements of gender homophily. In the extreme case, a field that initially has no women mentors and moves towards gender parity will show a change in homophily even if preferences for same-gender mentorship remain constant. We have therefore normalized measurements of homophily to the maximum attainable homophily given the gender balance of the population within a particular field at a particular time. Gender homophily remained positive across each field in each year even after normalizing to the maximum homophily attainable given the number of women participating in each field in each year (see Fig. 1B).

We also agree that correlations between gender and other variables, such as ethnicity and research interest, may influence gender homophily. We consider this issue in our discussion of the possible influence of the gender composition of subfields on homophily (pp. 19-20). As we note there, we find only a weak correlation between homophily and the fraction of women students and mentors within narrowly-defined research areas, suggesting that homophily is driven by choice of research supervisors or mentees rather than choice of research topic.

Unfortunately, we do not have data on trainee and mentor ethnicity and therefore cannot reach conclusions about its interaction with gender homophily. We have added a discussion point proposing that future research examine this question (lines 362-366).

A sense of affinity for the research group could also draw students to work with particular mentors. It would therefore be interesting to know whether there is more or less gender homophily in fields where mentoring is more one-on-one. Future research could also examine how gender homophily interacts with other demographic variables, particularly race and ethnicity.

**Reviewer #2:**

*Women STEM graduates are less likely than their male classmates to remain in the STEM workforce after graduation. This paper seeks to inform a potential explanation for this trend, documenting gender homophily in PhD mentoring relationships and describing differences by gender in PhD mentors' status as measured by their publications and citations (h-index), receipt of external funding, number of trainees, and institutional prestige.*

Major points

***R2.1.****. While the subject and motivation is certainly an interesting one, and the data effort is commendable, the research and its interpretation suffer from several critical drawbacks. First, as the authors note in the literature review section, female students are more likely than male students to cite their mentors as a source of psychosocial support. What's unclear from this descriptive evidence is the reason for this--that is, are female students more likely to need or seek out such support? Are female faculty more likely to offer or provide such support to their trainees, regardless of the trainee's gender? Whether through own preferences and desire to promote greater representation of women in STEM, or gendered expectations of female faculty, or some combination, this type of mentoring service can be very time-consuming and may detract from research productivity.*

While we argue that much of the impact of mentor gender can be explained by structural disparities in status, we agree that it would be helpful to understand how gender affects personal relationships and what impact this has on training outcomes. Because we do not have access to data on mentoring style or mentors’ service obligations, we cannot directly advance this discussion. However, we have expanded the Discussion to include additional context (lines 312-323):

Proposed mechanisms linking mentor and trainee gender to training outcomes include increased confidence and sense of belonging in a historically male-dominated field among women mentored by women [9]. Students have also reported qualitatively different support depending

on gender. Specifically, women graduate students are more likely to report benefiting from psychosocial support from a mentor during their Ph.D. training (such as providing emotional

support and taking an interest in the student’s personal life cf. [55]), whereas men are more

likely to report operational support (such as being involved in chairing a conference, collaborating on papers, or being recommended to colleagues, cf. [56, 57, 58]). It is not clear from this descriptive evidence whether such differences in support are driven by mentor preferences, trainee needs, or effects of gender stereotypes on trainee expectations of mentors [59]. Women faculty generally assume a greater share of academic service responsibilities than men [60], and differences in mentoring demands may add to this disparity.

***R2.2.*** *Second, and perhaps a greater concern, is potential misidentification and mischaracterization both of what's being measured and its implications. Because STEM fields have historically been more male-dominated, senior faculty members are more likely to be male. Senior faculty members also will tend to have higher status by all of the proposed measures (including prior number of trainees), with the most productive moving to higher-ranked institutions over time. Senior faculty members are also more likely to have built networks of tenured faculty at other PhD-granting institutions elsewhere to help in placing their students. The most obvious solution is simply to include years-since-PhD of the advisor in the regressions, which should be obtainable from the Proquest data.*

We agree that this is a reasonable interpretation of the data, and we considered it early in our analysis. As suggested by R2, we found that mentor seniority was correlated with training outcomes. However, when both seniority and the other success/resource variables were included in a single analysis, we found that the effect of seniority (years as independent researcher) and status (funding, h-index, etc.) on training outcome are independent. Thus, while mentor seniority also appears to impact training outcomes, it does not account for effects of status, which can vary among mentors with the same level of seniority.

To clarify this point in the revised text, we have incorporated mentor seniority into our main regression analysis. Mentor seniority was measured based on the date at which the mentor began an academic career. When data on mentor’s own graduate or postgraduate training was available, we considered the most recent training end date as the start of the mentor’s career. When no such data was available, the training start date was inferred from the date of the mentor’s first publication. This inference was based on a regression between training start date and first publication date for mentors in which both types of data were available. Because publication data was available for a much larger number of mentors than training data (*n*=43606 vs. 83682), this procedure substantially increased the coverage of mentor seniority data.

As suggested by R2, men mentors tended to have earlier career start dates than women mentors (mean = 1986 vs. 1989, *p*=1e-45, *n*=12731 mentors, *t*-test) as well as greater seniority at the date at which trainees graduated or ended their postdoctoral fellowship (mean = 20 vs. 22 years, *p*=9e-184, *n*=85400 training relationships, *t*-test). Greater seniority was positively correlated with various measurements of mentor status (see Fig. S8), as well as greater odds of trainee continuation (see Fig. 3).

However, contrary to the explanation suggested by R2, we still observe an association between mentor gender and trainee continuation after controlling for mentor seniority (Fig. 3), as well as a reduction in this effect after shuffling mentor status (Fig. 4). In a stepwise comparison, there was no consistent relationship between the change in effect of mentor gender after randomizing data for status and seniority (Fig. S9). Randomizing data for both status and seniority consistently resulted in a greater reduction of mentor-gender effects than randomizing either alone (Fig. S9).

The relationship between status, seniority, and gender is complex. It is likely that, in addition to the status variables explored in the manuscript, other status variables are correlated with seniority and associated with trainee success. However, our results indicate that gender differences in seniority do not entirely account for effects of gender inequities in status.

***R2.3.*** *Third, the "institutional bias" conclusion suggested in the abstract also has no direct evidentiary basis in the paper. If in fact the issue is that female faculty members have less access to resources, then the male and female trainees of those faculty members should be equally disadvantaged. This seems like something that could be tested using the authors' compiled data. Similarly, tests could be run comparing trainees of male and female faculty within a given field at a given university controlling for seniority.*

We have removed the reference to “institutional bias” in the abstract. The revised language reads (lines 23-26):

These results suggest that, in addition to other factors that limit career choices for women trainees, gender inequities in women mentor's access to resources and prestige contribute to women's attrition from independent research positions.

***R2.4.*** *Fourth, it's already been documented that female STEM PhDs are more likely to go into teaching-focused positions and institutions (e.g., liberal arts colleges). Individuals in these positions can have tremendous impact on undergraduates' persistence in STEM, but because lecturers, teaching professors, and faculty at non-PhD-granting institutions will not have PhD trainees of their own in the data, their outcomes are classified as "failure" in these models. Relatedly, gender-sorting across institutions means that availability of female mentors in one's field is not synonymous with availability of female mentors in one's PhD program.*

The manuscript now cites literature on the impact of early contact with women mentors in teaching-focused positions on undergraduates’ persistence in STEM. We have added a paragraph clarifying our motivation for focusing on continuation to mentorship in the discussion (lines 397-404):

An important limitation of the study is its focus on continuation to an academic job at a Ph.D.-granting institution as a measurement of training outcome. Continuation to mentorship is not the only successful outcome for trainees, but only one of many different and valuable paths that academic training makes possible. In particular, individuals that go into a teaching-focused job at a non-Ph.D. granting institution may have an impact on undergraduates’ persistence in STEM [9,10] or other fields. We focus on this path because of the role that graduate mentors play in defining research problems and training the next generation of faculty and the possibility that gender disparities in continuation to mentorship may be an indicator of sexism in academia.

***R2.5.*** *Fifth, from a methodological perspective, using first names to gender individuals (particularly trainees) is likely to lead to bias in the sample. The test check on the algorithm-assigned gender using photos is helpful, but it seems to be restricted only to those names that the algorithm could more definitively assign (p >= .7) as male or female. Since it is more common for historically male names to be repurposed as female names (e.g., genderize.io mistakenly assigns my female advisee Jordan as p=.97 male, and the author's algorithm would exclude both of my female advisees Amila and Bailey who have p=.68 female and p=.59 male respectively) this measurement error is likely to occur in only one direction: excluding women or misidentifying women as men.*

Thank you for pointing out this potential issue, which has motivated us to look more closely at the possibility of bias in measurement error. We have adjusted the thresholds for assigning gender to names to ensure equal probability of error for men and women and provided a more detailed breakdown of the results of the validation study. In addition, although unrelated to the current comment, we have improved the gender-classification algorithm by including information on location to account for geographic differences in the typical gender of names (see R3.5).

Our procedure for adjusting the threshold was as follows:

(1) For each probability *p* (0=woman, 1=man)*,* find the number of names, *n(p).*

(2) For each probability, calculate the number of names expected to be falsely assigned to each gender (e.g., for men expected error at probability *p = n(p) - p*n(p)).*

(3) Using the results from (2), calculate the cumulative probability of error if the threshold is set at each possible value of *p.*

(4) Choose a threshold to use for one gender.

(5) Using the results from (3), find the corresponding cumulative probability of error for one gender at this threshold. Choose the threshold for the remaining gender that gives an equal cumulative probability of error.

Following this procedure with a fixed threshold of *p* >= 0.75 for assigning male gender to names to men results in a slightly more conservative threshold for assigning female gender to names than that used in the manuscript (*p <=* 0.24 rather than *p* <= 0.25).

The results of a previous validation study included in a large, multi-national study of gender in science indicate that, at the population level, it is more common for algorithmic assignment of gender using names to misidentify men as women rather than women as men (Larivière et al. Nature 2013, Table S6, error rates: 13.3% for authors identified by algorithm as women vs. 1.7% for authors identified by algorithm as men). Our validation study shows comparable results, which we report in Table S3:

Finally, we examined the effects of adjusting the threshold on several key results and report them in Table S2. We compared the threshold of 75% used in the main text to a more liberal value of 60% and a more conservative value of 90%. For each threshold combination, we quantified (1) the median gender homophily across all data, (2) the marginal effect of trainee and mentor gender on trainee continuation to mentorship, and (3) the change in the effect of mentor gender after controlling for mentor status. In all cases, the effect of mentor gender on training outcomes is reduced after accounting for mentor status. Numerically, the reduction in the mentor gender effect was greatest when only *h*-index was considered (Fig. 4), but we report results for aggregate status in Table S2:

***R2.6.*** *In addition to the above drawbacks, even if we could definitively say that the outcomes of female trainees of female faculty are "worse" (in the sense of not continuing on to R1 PhD-granting academic jobs) than those of male trainees of male faculty, it's unclear what the policy implications and counterfactual would be. For example, we don't know whether reassigning female trainees to senior male faculty would actually result in better outcomes--among othe1r things, it's conceivable that female faculty are more successful in seeing their female trainees through to graduation who might otherwise have dropped out before earning the PhD, and the larger denominator due to retention of those marginally-attached scientists could drive some of the observed lower probability of remaining in R1 PhD-granting academia later.*

We hope the revisions based on feedback from all of the reviews helps clarify our conclusions that training outcomes for women are not affected directly by the gender of their mentor. (See also the reply to R3’s very detailed comments below.) Instead, we argue that trainees of women mentors are less likely to continue in independent research careers because women mentors, on average, have lower “status” than male mentors. We certainly do not advocate reassigning female trainees to senior male faculty. Instead, if we were to argue for any policy change, it would be to provide commensurate support for women mentors in academia, and have noted this in the discussion section (lines 340-341):

We view such results, like our own, as reason to work for equity in institutional support for women mentors in academia.

**Reviewer #3:**

*The work contains several minor and major issues that need resolved prior to publication. These are listed in the order that they appear in the manuscript.*

***R3.1.*** *On page 2, the authors should be precise in their use of the term productivity: there is a difference in the degree of disparities in production (i.e., the aggregate count of articles published) vs. productivity (the annually averaged count of articles published). In this sentence, it also seems to imply that citation and levels of funding are subsets of productivity.*

Following other revisions, we have moved this sentence to the results and revised it as follows (lines 295-297):

These [gender disparities] include lower production for women researchers, as measured by number of publications [11, 14, 6], as well as disparities in rate of citation [39], and levels of funding [40, 41, 42, 43, 44].

***R3.2.*** *The authors provide scant literature on caregiving as an explanation of the productivity puzzle. They may want to consult the work of Frank Fox and the recent work by Morgan et al., among other literature. The current paragraph implies that all cited works refer to differences in partner employment, rather than child- or elder-care.*

We have separated the citations to indicate which refer to differences in partner employment, and added more detail on studies of caregiving (lines 305-311):

Effects of bias may be amplified by constraints and cultural expectations related to family life, particularly childcare, which impact women more than men researchers, especially in early and mid-career stages [52, 27, 14]. Recent longitudinal studies indicate that parenthood impacts women’s participation in the STEM workforce more than men [37] and accounts for a large share of gender differences in research production [53]. Geographical constraints due to partners’ professional employment may also impact women in academia to a greater degree than men [54].

***R3.3.*** *There is some misalignment between the concluding paragraph on page 4 and the abstract. I would urge the authors to utilize the language on the results in the former, as it appears more accurate and less likely to be misleading to journalists. The current abstract may introduce errors in the dissemination of the results. For example, it states: "Trainees of women mentors are less likely to continue into independent research careers that trainees of male mentors." However, the paper does not measure whether they become researchers, but whether the mentees become mentors. This is a very important distinction.*

We have incorporated a more accurate exact statement of the results into the abstract (lines 8-13):

Student and mentor gender are both associated with differences in rates of student’s continuation to independent mentor positions of their own. Although trainees of women mentors are less likely to become mentors than of men mentors, this effect is reduced substantially after controlling for several measurements of the mentor’s status.

***R3.4.*** *The authors should provide more details of the validity of the Academic Family Tree. Several crowdsourced academic genealogy projects are highly heterogeneous in the degree to which the relationships indicated were formally doctoral advisors. In many cases, these databases contain links to other formative relationships (in some cases, referencing dissertation advisors before dissertation advisors existed (see, e.g., the Mathematics Genealogy Project). For more on these types of limitations, see the chapter by Sugimoto in the Cronin and Sugimoto edited work (Beyond Bibliometrics).*

Given that the data was limited to the period 2000 to 2015, we are not concerned about heterogeneity due to historical change in the nature of mentoring relationships. We have provided more detail on the validity of the Academic Family Tree data in the methods section (lines 410-421):

The AFT derives information on training relationships from two sources: crowdsourced (i.e., user-provided) data and ProQuest’s collection of dissertations and theses. In a previous study, a portion of the crowdsourced data was validated by comparison with data on formal mentoring relationships indicated on faculty web pages [21]. To limit heterogeneity in the type of mentoring relationships included [75], ProQuest data was limited to records of dissertations that resulted in doctoral degrees within a recent time period (2000 to 2015) and listed the dissertation advisor. When the name and institutional affiliation of an advisor listed in ProQuest matched the name and field of study of a mentor included in crowdsourced data, the two were considered to refer to the same person. If no name match was found at any institution, a new node was added for the advisor. If a name match was found at another institution, the training relationship was not added until it could be reviewed manually as being a match to an existing mentor or a new mentor.

***R3.5.*** *The authors may want to utilize more robust gender disambiguation algorithms that take into account not only the first name, but also the last name and nationality of authors. See, e.g., Ludo Waltman's recent work on this, comparing several approaches.*

For the initial manuscript, we used gender probability estimates based on usage data pooled across multiple locations. However, it is possible that this method does not provide accurate results for names with strong geographic variation in gender (e.g., “Robin” is typically a woman’s name in the U.S. and a man’s name in the U.K.). To refine our estimates, we therefore limited data on which gender estimates for each individual to usage data drawn from the country in which each individual’s academic institution is located. When no location information was available, we used gender estimates based on all available data. We found a strong correlation between gender probabilities estimated with and without location information (Pearson’s correlation coefficient = 0.99, 555672 researchers). We also observed a strong agreement between the previous and new methods reached after thresholding to remove ambiguous gender probabilities (see R2.5) and dividing data into two gender categories (fraction of data with different classifications under previous and current method: 0.2%, 530008 researchers).

Waltman et al. considered data on last names for researchers in a limited number of Eastern European countries in which surnames use gendered suffixes. Given that only 0.01% of researchers in our dataset are based in institutions in these countries, we did not consider last names in our analysis.

***R3.6.*** *This statement is unclear to me: "The fraction of women mentors in each career-start cohort increased over time, and remained stable from 2000 to 2015."*

We have rephrased the sentence and context to read (lines 141-150):

We quantified the fraction of women mentors, fraction of women students, and homophily as a function of both mentor’s career start date (1970 to 2015) and student’s graduation date (2000 to 2020). The fraction of women beginning careers as mentors increased over time from 1970 to 2015 (Fig. S7A). After controlling for mentor’s training end date, there was no relationship between the fraction of women mentors and trainee’s training end date. This result suggests that the increase in women mentors was not driven by mentor retirements between 2000 and 2015. The fraction of women students trained by the mentors also increased between 2000 and 2020 (Fig. S7B). The decrease in homophily between 2000 and 2020 was related to time (i.e., graduation year), but not mentors’ academic age (Fig. S7C).

***R3.7.*** *The indicators of resources seem to be very heterogeneous. What is the justification for the inclusion of the h-index as a "resource"? Or the trainee count? More justification should be provided for this operationalization of resources. (Also, what is the justification for using the h-index rather than just using publications and citations separately?)*

We agree that the term “resources” is confusing and a more appropriate term for these indicators is “status”.

We attempted to define a wide-ranging set of metrics that capture the mentor’s status within the field relative to other mentors. Some of these metrics, such as funding, are a more operationalization of resources than others. Trainee count was included because it offers a rough proxy for lab size, which is related to a mentor’s ability to obtain funding and to carry out ambitious projects. We included h-index because it is a widely used and simple metric that summarizes a mentor’s entire publication history. Other metrics are possible, but given the strong correlations between them (see Fig. S8), we expect that using different ones would not substantially impact the results.

We have included text clarifying this point (lines 164-177):

We hypothesized that if men and women mentors differ in status (defined as access to funding, labor, and prestige markers such as citations) due to gender bias, these disparities might lead to differences in trainees’ continuation to academic mentorship roles. We therefore compiled several widely used metrics to quantify mentor’s status: h-index (a measurement of citation rate and publication production [29] based on data from the National Library of Medicine and Semantic Scholar), trainee count (total number of Ph.D. students and postdocs mentored, a metric closely related to laboratory size [3]), the rate of funding granted by the US governmental agencies National Science Foundation (NSF) and National Institutes of Health (NIH), and the rank of the mentor’s academic institution in the Quacquarelli Symonds World University Rankings, an annual assessment that heavily weights the institution’s reputation among academics. Funding rate, h-index, and trainee count were all positively correlated with one another, and negatively correlated with work at a low-prestige institution, suggesting that all four metrics measured a common trait of “aggregate status” (Fig. S8).

***R3.8.*** *The authors conflate sex and gender throughout (switching between male/female and men/women).*

Thank you for highlighting this point. To indicate that we are referring to gender, we have avoided using the terms “male” and “female” and instead used the adjectival forms of “men” and “women” (*e.g.,* “men mentors” and “women mentors” rather than “male mentors” and “female mentors”).

***R3.9.*** *The authors discuss the preferential selection of men by elite scientists, but fail to connect this with the larger literature on the Matthew effect and cumulative advantage.*

Thanks for pointing this out, we now cite the relevant line of research when we introduce the analysis of elite scientists (lines 233-236).

It is well-established that research communities are biased to value the output of researcher who have established a high profile [Merton 1968]. Moreover, it has been reported that men mentors in this group train fewer women than their peers, possibly exaggerating the impact of homophily in this group [Sheltzer et al 2014].

***R3.10.*** *The most novel contribution of this paper is the focus on the relationship between mentor status and advancement as students as trainees, which suggests that it is not the attribute of the gender of the mentor that is important, but the fact that gender is associated with differential levels of symbolic capital. However, this is given short-shrift in the discussion and the results are written ambiguously across the manuscript, and often focusing on the disadvantage of training with women mentors. Take, e.g., this sample of statements:*

**	Trainees of women mentors are less likely to continue into independent research careers that trainees of male mentors.*
**	Our results do not suggest a penalty for the careers of women than work with women PhD mentors.*
**	Mentor gender effects on retention are not statistically significant for women graduate students.*
**	Higher mentor status was associated with greater rates of trainee continuation.*
**	Controlling for mentor status reduces the effect of mentor gender on trainee retention by up to 47%.*
**	Controlling for mentor's academic achievements reduces this disparity, but has a minimal impact on the disparity between male and female trainees.*
**	Our general finding that differences between male and female mentors are reduced after controlling for access to resources.*
**	Women graduate students and postdocs have lower continuation rates than men, even after controlling for mentor gender and mentor's academic achievements.*
**	Mentor-gender effects did not reach significance among all subsets of the data.*
**	Gender groups are also associated with differences in mentoring outcomes.*
**	Mentors that received a prestigious award tended to train more male students.*
**	Homophily is unrelated to mentor achievements.*
**	Gender homophily occurs among all general research areas.*

*Each of these statements may be accurate, when listed with all their caveats and context. However, taken together, they create considerable confusion on the main findings of the manuscript. Furthermore, taken independently, they could have strong potential for misinformation. I would strongly urge the authors to carefully read through the manuscript and focus on precision in language and clarity in organization. This is important work, but must be done robustly and communicated precisely.*

Thank you for drawing our attention to this issue. We appreciate that this study reports several analyses that build towards nuanced conclusions. Recognizing that not all readers will read the entire manuscript with close attention, we have revised the text throughout the manuscript to state the results with less ambiguity, including all the specific points identified above.

The manuscript contains two analyses of mentor gender and training outcomes. The first shows that mentor gender is associated with differences in trainee continuation. The second demonstrates that this apparent association is reduced after considering mentor status. Therefore, as R3 accurately summarizes, “it is not the attribute of the gender of the mentor that is important, but the fact that gender is associated with differential levels of symbolic capital.” Much of the confusion created by the quoted excerpts is a result of juxtaposing descriptions of these two analyses without information on their relationship to each other. We have therefore moved the first analysis to a supplementary appendix and revised the abstract and discussion to emphasize the result of the second analysis.

Similarly, the manuscript reaches a nuanced conclusion about the relationship between gender homophily and mentor status. Gender homophily is unrelated to *most* metrics of mentor status we consider. However, mentors that are recipients of prestigious awards train significantly more men than high-status mentors that are not prizewinners. In addition to many small changes noted above, we have modified the following key statements to emphasize this conclusion:

“Mentors that received a prestigious award tended to train more male students.” TO “Our results thus build on the finding that elite men mentors in the life sciences employ fewer women than their colleagues [20], suggesting that it does not generalize to other indicators of prestige (as it would if we had found a consistent decrease in the percentage of men students across success levels).” (lines 368-373)

“Homophily is unrelated to mentor achievements.” TO “We show that homophily is unrelated to markers of mentor status, with the exception of receipt of a prestigious award.” (lines 367-368)
