# Supplementary material for: Impact of gender on the formation and outcome of formal mentoring relationships in the life sciences
Source: PLoS Biol. 2022 Sep 8;20(9):e3001771. doi: 10.1371/journal.pbio.3001771 (PMC9455859; doi:10.1371/journal.pbio.3001771)
Supplement: Attachment — Submitted filename: r2r rev2.docx [file pbio.3001771.s018.docx]

We thank the editor and reviewers for additional constructive feedback on this manuscript (PBIOLOGY-D-22-00842R1). The revised manuscript takes their comments into account and, we believe, is now clearer in how it addresses challenging and complex questions around gender in mentorship. Point-by-point replies to each comment are provided below. All changes are indicated in a redlined version of the manuscript, included with this resubmission.

Reviewer #1:

*In the response to my comments, the authors have provided analyses or evidence to address my concerns in previous rounds. The current manuscript is a clear improvement. Here, I have several suggestions as below:*

*1. The authors should take care not to generalize beyond what they are able. The title, for example, should clearly stipulate that this is focused on life sciences.*

We have revised the title to reflect our focus on formal academic mentorship in the life sciences: “Impact of gender on the formation and outcome of formal mentoring relationships in the life sciences”.

*2. In addition, I am also interested to know whether the main findings generalize to other disciplines, for example social sciences and engineering. If data are limited to answer this question, the authors should discuss this limitation in the final draft.*

Our findings on homophily generalize across fields (Fig. 1, S4, S5, S6, Table S1), as do some of our findings on academic continuation and gender (Appendix, Fig. S11). We have limited our analyses that include mentor status to the life sciences. We clarify the reasons for this focus in the revised text:

We limited our analysis of mentor status to the life sciences. Sampling was more complete in these fields for two reasons. First, data on publication and funding was drawn from sources specific to this field (including funding data from the National Institutes of Health and publication data from the National Library of Medicine). Second, because the Academic Family Tree began as an effort to crowd-source the academic genealogy of neuroscience, its sampling of mentorship data is most dense for the life sciences. (Results, p. 5, lines 168-174)

We offer evidence that gender-based disparities in the status of mentors in the life sciences contribute to disparities in the rate at which their trainees continue to academic mentorship roles. Because our sources for data on citation, funding, and mentor receipt of an award are most complete for the life sciences, our results on mentor status are limited to this field, unlike our results on homophily and the leaky pipeline phenomenon, which spanned multiple fields. (Discussion, p. 9, lines 302-307)

*3. A student can have multiple mentors in her/his career. For example, student A has mentors M1, M2 and M3. A-M1 is a woman-woman pair. A-M2 is a woman-man pair. And A-M3 is also a woman-man pair. A's discontinuation can be affected by all these three mentor-mentee relationships. I was wondering whether mentor fixed effect is taken into consideration. If existing data cannot support such kind of analysis, please include a limitation discussion.*

This is an interesting question. The number of trainees with status data available for both graduate and postdoctoral mentors was too small to support a complete analysis. However, we were able to fit a simpler model that incorporated the gender and temporal variables for trainee-mentor triads, and we believe this analysis addresses the question. For this group, only the gender of the second, postdoctoral mentor is correlated with trainee continuation. This new result is included in the appendix:

The above data focused on the outcome of Ph.D. training only. However, associations between mentor and trainee gender and trainee continuation were present at both the transition from Ph.D. to postdoc and postdoc to mentor (Fig. S11G). To test the joint influence of multiple mentors on trainee continuation, we identified the group of trainees with records of both graduate and postdoctoral training in Academic Family Tree, and fit logistic regression models that included temporal controls (training date and mentor seniority), trainee gender, and the gender of the graduate mentor, postdoctoral mentor, or both. Interestingly, in this case only the gender of the postdoctoral mentor was associated with training outcomes (n = 18474, beta = 0.261, p = 0.0089 [postdoctoral mentor], beta = 0.121, p = 0.398 [graduate mentor]). We observed the same pattern if we focused only on life sciences data (n = 11201, beta = 0.374, p = 0.007 [postdoctoral mentor], beta = 0.159, p = 0.256 [graduate mentor]). This difference suggests that among trainees that continue to a postdoc, the gender of the postdoctoral mentor exerts a stronger influence on continuation than the gender of the graduate mentor. (Appendix, p. 2)

Reviewer #2:

*The authors' efforts to address reviewers' comments on the previous version are commendable, and the manuscript has certainly improved, including in its exploration and discussion of limitations. Nonetheless, I think there are significant issues remaining in terms of how the research and its findings are presented, starting with the abstract. I highlight some examples below, but recommend re-framing the discussion throughout the paper along these lines as well.*

*"Despite increasing representation in graduate training programs, a disproportionate number of women leave academic research before obtaining an independent position."*
*"Although trainees of women mentors are less likely to take on independent research positions..."*

*The outcome stated ("independent [research] position") is not what is studied in this paper. Many of us (consider your readers...) are independent academic researchers by any measure, in tenure-track faculty positions, PIs on NIH and NSF grants, mentoring many students on research including on their theses and as RAs on our sponsored research projects, but are nonetheless at institutions that do not award doctoral degrees. (Furthermore, not all PhD-granting department faculty who formally supervise doctoral theses are truly mentors, and many good mentors to a doctoral student may not be their formal PhD supervisor.) The outcome measured in this paper is whether an individual goes on to supervise (as formal advisor) a completed PhD dissertation, or possibly a postdoc. As the authors suggest, PhD supervision is an important measure as PhD advisors are training the next generation of academic faculty researchers and scientists. But the language used in the title, abstract, and throughout the paper should reflect what this study is actually examining.*

We agree that it is important to avoid misunderstanding of this outcome variable. In the revision, we have consistently adopted the term “academic mentor” to refer to the type of supervisory role the reviewer describes. We define the term in the introduction: as “a position that allows one to serve as a formal supervisor to Ph.D. students and/or postdocs" (p. 4, lines 78-79). We also emphasize this aspect of the outcome variable at several points in the text, including the two highlighted by the reviewer (*italics* added here to indicate specific changes):

Despite increasing representation in graduate training programs, a disproportionate number of women leave academic research *without obtaining an independent position that enables them to train the next generation of academic researchers*. (Abstract, p. 1)

Student and mentor gender are both associated with differences in rates of *student’s continuation to positions that allow formal academic mentorship*. Although trainees of women mentors are less likely to take on *positions as academic mentors* than trainees of men mentors, this effect is reduced substantially after controlling for several measurements of mentor status. (Abstract, p. 1)

Gender-associated differences in mentor status may in turn impact the ability of trainees to perform high-impact research and thus reduce the likelihood of *continuing on to formal academic mentorship roles*. (Introduction, p. 2, lines 25-28)

Consistent with previous investigations into the attrition of women across the academic career track (sometimes known as the "leaky pipeline") [26, 27, 14, 28], our results show that the proportion of women in social science and STEM fields is lower at progressively later stages of the academic career track, from graduate student to postdoc to *academic mentor* (Fig. S12A). This result indicates the population of *academic mentors* remains skewed towards men, even in research areas with student populations close to gender parity. However, it does not in itself indicate whether women graduate students *continue on to academic mentorship positions* at the same rate as men graduate students. In addition, it does not indicate whether structural gender biases that affect women as mentors indirectly affect *retention of their students in academia as formal mentors*. (Results, pp. 4-5, lines 138-147)

Due to our strict definition of continuation as progress to *academic mentorship*, it is likely that the continuation rates reported here (see Supplementary Data, “Relative Continuation Rate Analysis”) underestimate the actual proportion of trainees that remain in academia. (Results, p. 5, lines 180-183)

Gender groups are also associated with differences in mentoring outcomes, as measured by trainees' continuation to positions that allow for *formal supervision of doctoral students and postdocs*. (Discussion, p. 8, lines 266-269)

These findings support a model in which mentors' access to funding and labor, as well as prestige markers such as citations, are distributed unevenly by gender, and in turn affect trainees' *retention in academia as formal mentors*. (Discussion p. 8, lines 275-277)

Our finding that rates of continuation to academic mentorship are lower for women graduate students and postdocs replicates prior research that has identified post-graduate career transitions as points of women's attrition from academia, *although we note that there is a distinction between acting as formal mentor, holding a tenure-track position, and participating in academic research in any capacity* ... (Discussion, p. 8, lines 281-285)

We offer evidence that gender-based disparities in the status of academic mentors in the life sciences contribute to disparities in the likelihood with which their trainees continue *to academic mentorship roles.* (Discussion, p. 9, lines 302-304)

We have incorporated the reviewer’s feedback regarding the range of career paths into our discussion of the study’s limitations:

An important limitation of this study is its focus on placement in positions that enable formal supervision of doctoral students and postdocs as a measurement of training outcome. Formal academic mentorship does play an outsized role in shaping research communities, and thus merits special attention. At the same time, taking on this mentorship role is only one of many different and valuable outcomes that academic training makes possible. Individuals that go into *a teaching-focused job at a non-Ph.D. granting institution* may have an impact on undergraduates’ persistence in STEM [9,10] or other fields. Trainees may also directly advance scientific knowledge through work in industry or conducting and supervising research at *non-Ph.D. granting academic institutions*. We focus on doctoral and post-doctoral supervisors because of the role they play in defining research problems and training the next generation of faculty, as well as the possibility that gender disparities in this specific trajectory may be an indicator of more widespread sexism in academia. (Discussion, p. 11, lines 434-446)

*Minor point: I would remove the "multidisciplinary database" references in the abstract and the text since you're focusing now on a single discipline. Similarly, in the introduction, the discussion about lack of literature comparing across fields seems out of place since the paper's looking at a single field.*

We agree that it is important to avoid misunderstanding about which of our findings apply to multiple disciplines, and which apply to life sciences. Because our most developed set of results concerns life sciences, we have eliminated the phrase “multidisciplinary database” in the abstract and highlighted the focus on life sciences in the title and abstract.

The text’s reference to the lack of literature comparing across fields concerns studies of homophily:

Surprisingly little is known about the drivers that influence homophily. In particular, it is currently unknown if there are field-level differences. (Introduction, p. 2, lines 34-36)

While some analyses focus on life sciences, we do examine gender homophily in graduate training across multiple fields and subfields and find it is ubiquitous but has been decreasing over time in several research areas (Fig. 1, S4, S5, S6, Table S1). In addition, we analyze the association of mentor gender and trainee progression to academic mentorship in multiple fields and find consistent trends (Appendix, Fig. S11). References to the lack of comparative studies of homophily and the multidisciplinary nature of the dataset are therefore necessary to motivate a large portion of the analysis and results.

The current draft has indeed limited the discussion of mentor status and its interaction with gender and training outcomes to the life sciences. This is because our sources of data on mentor status are the most complete for this area. Both the text and figures clearly designate which findings are limited to the life sciences: note, for instance, the heading “Life Sciences” above Figs. 2, 3, 4, and 5, the references to the life sciences in the legends of Figs. 2, 3, 4, and 5, and the following text from the introduction (italics added):

We find that gender homophily in graduate training is ubiquitous *across fields*, despite differences in the proportion of women students and faculty. *Focusing on data from the life sciences*, where sampling of mentorship data is more exhaustive, we find that both student and mentor gender are associated with different rates of trainee advancement to an academic mentorship role (i.e., a position that allows one to serve as a formal supervisor to Ph.D. students and/or postdocs). However, the effects of mentor gender on this training outcome are substantially reduced after controlling for several measurements of the mentor's status. Gender homophily *in the life sciences* is generally unrelated to mentor status. (Introduction, pp. 2-3, lines 50-58)

*Despite that, the broad field of biological sciences includes many, many subfields with different publication and citation practices, so this focus on one field doesn't fully address the possibility of gender-sorting on research topics and differences across, say, immunology versus ecology.*

We agree that our results motivate future studies that include more fine-grained analyses of research communities. We discuss the need for such studies in relation to homophily (Discussion, “Homophily in academic mentorship”, p. 21, lines 373-396). The issue raised by the reviewer is whether statistical trends observed at the level of the biological sciences would disappear upon division into subfields or research topics. This could occur if three conditions are met:

(1) Topic A attracts more men mentors and trainees than Topic B.
(2) Mentors studying Topic A rank higher on status markers than mentors studying Topic B.
(3) Trainees studying Topic A are more likely to continue to academic mentorship roles than trainees studying Topic B.

Such a pattern would suggest that the allocation of prestige and resources across research topics is an important mediator of gender differences in training outcomes. It would also raise further questions about the role of gender bias in determining such allocation. However, the results would still be consistent with the trends we have observed at the level of individual mentors and trainees in the life sciences.

Given that it would require substantial further analysis and additional data to settle these questions, we believe that, as with more detailed analyses of homophily, the question is best left as a topic for future research. We have therefore noted this in the discussion:

Future research could also examine whether subfields or research topics in the life sciences differ in the relationship between mentor gender, mentor status, and trainee continuation. (pp. 9, lines 347-349)

*Counting both funding from federal agencies and number of trainees (most of whom would be supported on that funding) as status measures seems a bit like double-counting, since the number of graduate students in these fields scales as a direct function of federal research and trainee funding (see e.g. Blume-Kohout and Clack, https://journals.plos.org/plosone/article?id=10.1371/journal.pone.0082759 ). Including total trainees as a predictor of having a trainee be successful seems subject to reverse causality.*

We agree that the observation that funding level and trainee counts are correlated at a macroscopic level, one might also expect them to be correlated within research groups. Indeed, we demonstrate correlation in Figure S8. Our analysis considers these variables as alternative measures of status. The dimensionality reduction method used to compute aggregate status does not simply add them but instead combines them in a weighted sum to produce a single, more reliable measure, so we believe this avoids a problem of double-counting.

However, we do not agree that the use of trainee count as an independent variable in models predicting trainee continuation rate creates a problem of reverse causality. The dependent variable, training outcomes is measured per trainee. Thus, while a larger lab may be more likely to overall produce more trainees who move on to academic mentorship, the probability of a single trainee continuing does not depend numerically on the number of other trainees in that lab.

We chose trainee count as a status metric specifically because it has been shown empirically that trainees from large research groups are more likely to continue on to academic mentorship positions (Malmgren et al. 2010, Lienard et al, 2018).

Based on this logic, we expected status variables to predict continuation rates. By incorporating status variables into the multivariate regression, we sought to test whether those benefits could explain the apparent advantage conferred by training with a man mentor.

A related concern, to which we have given substantial consideration, is the possibility that long-term trends in global levels funding could be confounded with changes in trainee continuation rates over time. We believe that we have addressed concerns about long-term trends with the inclusion of trainee end date and mentor seniority variables in the regression analysis (Fig. 3).

We now clarify the point that inclusion of training end date in the regression analysis accounts for long-term global trends:

Including training end date as an independent variable accounted for long-term changes in the number of trainees, status variables and continuation rates. (p. 6, lines 193-195)

*"Thus the effect of mentor gender can be explained at least partially by gender disparities in social and financial resources available to mentors."*

*There's no evidence of gender disparities in NIH funding outcomes conditional on applying (see the series of articles by Donna Ginther and colleagues, e.g., https://journals.plos.org/plosone/article?id=10.1371/journal.pone.0205929), and with federal agency funding the only measure of financial support in this study, I don't see evidence to support the conclusion that women scientists should get more financial support.*

This is an important reference, suggesting that the NIH review process may not be driving the gender disparities that we observe in mentor status. It also highlights the attention that has already been given to these challenging issues. This result suggests that the gender disparity in mentor status results from other factors than study section bias. Given that overall levels of funding do show a gender disparity in our analysis (Fig. 3), the cause must lie either in the frequency with which men and women apply for funding or the size of the grants they are awarded. We have added these points in the text:

Given the many factors that can lead to gender disparities, it is unlikely that equity can be achieved with a single change. For example, analysis of NIH data suggests that outcomes of grant review are not biased against women applicants [Ginther et al. 2016]. Still, a disparity in funding levels persists, which must reflect differences in the rate at which women apply for funding or in the size of awards. (p. 10, lines 362-366)

*Given the option, wouldn't all PhD students prefer the advisor who is best positioned to make them successful? Should institutions assign more PhD students with institutional or fellowship funding to women faculty (regardless of the student's preference), to increase women faculty members' status? Evidence indicates biomedical sciences PhD students with traineeship or fellowship funding are less likely to take scientific research jobs at graduation, compared to students who were primarily funded as research assistants (see https://www.sciencedirect.com/science/article/pii/S004873331630035X ), so it doesn't seem like this would work.*

*I'd encourage the authors to reconsider what policy implications can credibly be drawn from this research.*

The reviewer highlights complexities both of understanding the role of gender in mentorship outcomes and of how to address gender inequality. A general implication of the current study is that if disparities in mentor status are reduced, this will also reduce disparities in trainee outcome. Because the many status variables co-vary, it is not clear if one of them is the primary driver. However, among status variables analyzed, h-index was the factor most strongly associated with continuation. Thus, efforts that lead to equalization of metrics like h-index may be important. We have incorporated this point into the Discussion:

In this study, the status variable that accounted best for the disparity in mentorship outcomes was h-index. While h-index can be criticized as overly narrow in its scope, this observation is consistent with the idea that a researcher with high publication impact is likely to be well known and respected within their field. By this logic, investment by universities and professional societies in programs that enable women mentors to increase the impact of their work in ways that increases metrics such as h-index may also benefit trainees seeking positions as academic mentors. (p. 10, lines 366-373)
